# Transferrin-modified multicomponent liposomes encapsulating paclitaxel-loaded *β*-elemene microemulsion enhance therapeutic efficacy in non-small-cell lung cancer

**DOI:** 10.1016/j.ijpx.2026.100488

**Published:** 2026-01-12

**Authors:** Yunyan Chen, Ziwei Zhang, Rui Xiong, Yuqing Cao, Qian Liu

**Affiliations:** a School of Pharmacy, Wannan Medical College, Wuhu 241002, China; b The Affiliated Brain Hospital of Nanjing Medical University, Nanjing 210029, China

**Keywords:** PTX, *Β*-Elemene, Liposomes, Tumor target, NSCLC

## Abstract

To achieve efficient accumulation and facilitate profound penetration of anti-tumor agents within neoplastic tissues stands as one of the most critical determinants influencing the efficacy of anticancer therapies. Herein, a multicomponent-based liposomes (Tf-PEM/L) by transferrin-modified encapsulating paclitaxel (PTX)-loaded *β*-elemene microemulsion (PEM) was fabricated, demonstrating significantly enhanced therapeutic efficacy against non-small cell lung cancer (NSCLC). Leveraging the synergistic mechanism of transferrin-mediated active targeting coupled with the enhanced permeability and retention (EPR) effect, Tf-PEM/L demonstrates a pronounced propensity for efficient and substantial accumulation at the tumor site. Following accumulation, the subsequently released PEM enables highly efficient deep penetration within tumor tissue, thereby achieving favorable anti-tumor therapeutic efficacy. Characterization of Tf-PEM/L revealed a mean particle size approximately (144.76 ± 9.34) nm, while the zeta potential exhibited a measurement of (−12.52 ± 0.28) mV. Notably, the transmission electron microscopy (TEM) images revealed the small-sized PEM were encapsulated within large-sized liposomes. In vitro cytotoxicity assays demonstrated that Tf-PEM/L elicited synergistic antitumor effects against A549 cells, underscoring its combinatorial therapeutic potential. In vivo studies, Tf-PEM/L demonstrated exceptional tumor-targeting capabilities as evidenced by quantitative biodistribution analyses. Moreover, Tf-PEM/L exhibited superior antitumor efficacy with tumor inhibition rate of (81.36 ± 3.87)% while markedly attenuating systemic toxicity, positioning it as a promising therapeutic strategy for NSCLC. Collectively, the Tf-PEM/L represents a promising targeted therapeutic strategy for NSCLC, with enhanced efficacy and safety profiles.

## 1 Introduction

Lung cancer represents one of the most widespread and formidable malignant neoplasms on a global scale, characterized by a notably high incidence rate and an alarming upward trajectory in mortality, necessitating the urgent development of novel and effective treatments (Lardinois, 2012). Advanced stages of lung cancer frequently entail the occurrence of brain metastasis, with an alarming 20% to 40% of patients diagnosed with non-small cell lung cancer (NSCLC) exhibiting such neurological involvement (Hou et al., 2024). The poor blood-brain barrier (BBB) permeability of anti-tumor drugs contributes to the poor anti-tumor efficacy of standard chemotherapeutic drugs (Gerstner and Fine, 2007). Compared to the conventional chemotherapy drugs, nanodrug delivery systems can reduce the toxicity of anti-tumor drugs while simultaneously enhancing drug accumulation at tumor sites through sophisticated tumor-targeting mechanisms (Kong et al., 2023; Andreea et al., 2023). Liposomes have been studied as alternative chemotherapy options due to significant advantages in reducing systemic cytotoxicity, there are many commercial products listed nowadays with magnificent market application prospect (Tian et al., 2015).

Paclitaxel (PTX) is derived from *Taxus brevifolia* bark, which is widely used for treating lung, breast, and pancreatic cancer (Alavi and Nokhodchi, 2022; Cai et al., 2020). However, its application in advanced NSCLC patients is challenging due to low tolerance and toxic side effects to chemotherapy during treatment leading to unsatisfactory clinical outcomes (Baez Gonzalez et al., 2023; Subedi et al., 2024).

*β*-elemene is extracted from *Curcuma zedoaria (Christm.) Rosc*, represents a significant component of the terpene family. *β*-elemene is employed as an adjunctive therapy in the conventional treatment of NSCLC due to its multifaceted mechanisms of action, which include effectively reversing drug resistance, inducing apoptosis in tumor cells, and inhibiting angiogenesis. (Han et al., 2022; Xu et al., 2024).

The transferrin receptor (TfR) is a cell surface protein that is significantly overexpressed on the membrane of NSCLC cells, playing a critical role in mediating cellular iron uptake and supporting tumor progression, thereby rendering TfR an enticing target for the development of specific antibodies. His high TfR expression enables specific binding to Tf-modified nanoparticles, which in turn enhances the active internalization and uptake of nanocarriers by NSCLC cells. Modifying nanoparticles' surface with tumor-targeting ligands can improve drug accumulation at tumor tissue effectively by reducing chemotherapy toxicity while also aiding in crossing the BBB for treating NSCLC brain metastasis (Itoh et al., 2023; Annie et al., 2022).

**Scheme 1:**
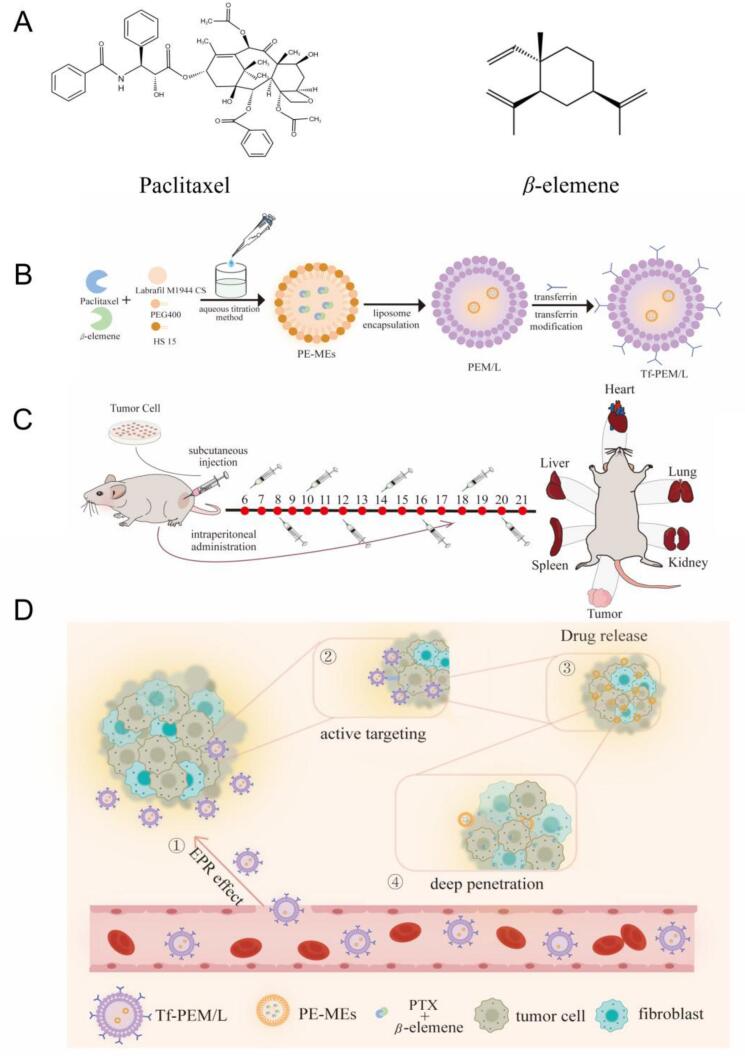
(A) Chemical structures of Paclitaxel and *β*-elemene. (B) A Paclitaxel -loaded *β-*elemene microemulsion lipid complex with transferrin modification (Tf-PEM/L), paclitaxel acted as the main antitumor component and *β*-elemene was used in the oil phase and acted as an antitumor ingredient, and Tf as an active targeting ligand was conjugated on the surface of Tf-PEM/L. (C) and (D) Construction of tumor models and active targeting process of drugs in vivo.

Based on our previous report, we have fabricated a PTX-loaded *β*-elemene microemulsion (PE-MEs) (Chen et al., 2024a). By fabricating the PTX-loaded *β*-elemene microemulsion lipid complex with transferrin modification, Tf-PEM/L can target the tumor tissues efficiently with transferrin modification and EPR effect, and meanwhile the small-sized PE-MEs was released subsequently to achieve deep penetration at the tumor sites (Scheme 1). This study presents an innovative approach to enhancing drug delivery efficiency and anti-tumor efficacy by leveraging the tumor-targeting and deep penetration of tumor sites by Tf-PEM/L. The integration of these features offers a novel paradigm for multi-component nano drug delivery systems derived from traditional Chinese medicine (TCM).

## 2 Materials

Paclitaxel (98.0%), Polyethylene glycol 400(PEG 400), CHCl_3_ and CH_3_OH were all sourced from China National Pharmaceutical Group Co., Ltd. (Shanghai). *β*-elemene (98.0%) was graciously supplied by Hubei Yunmei Technology Co., Ltd. (Hubei). Dipalmitoylphosphatidylcholine (DPPC), 1,2-Distearoyl-sn-glycero-3-phosphoethanolamine-N-[methoxy(polyethyleneglycol)-2000] (DSPE-MPEG_2k_) and S-lysophosphatidylcholine (S-lysoPC) were furnished by AVT Co., Ltd. (Shanghai). HS15 was offered by BASF Co., Ltd. (Ludwigshafen, Germany), and Labrafil® M1944 CS was furnished by Gattefosse. Dulbecco's Modified Eagle Medium (DMEM**)**, Fetal bovine serum (FBS), phosphate-buffered saline (PBS) and EDTA-0.25% trypsin solution were procured from Gibco Co., Ltd. (California, USA). A549 cells were acquired from the Chinese Academy of Sciences (Beijing). All other chemicals and reagents utilized in this study adhered to analytical grade standards unless otherwise specified.

## 3 Methods

### 3.1 Animals

Male Nude mice (BALB/c, 25 ± 2 g) were procured from Jiangsu Huachuang Cigna Pharmaceutical Technology Co., Ltd. Prior to the study, the mice were housed in a controlled environment with a 12-h light-dark cycle and provided with standard laboratory diet and water ad libitum for a minimum acclimatization period of one week. All experimental protocols were reviewed and approved by the Animal Experiment Ethics Committee of Wannan Medical College (Wuhu, China), ensuring compliance with ethical guidelines for animal research (WNMC-AWE-2024094).

### 3.2 Preparation and characterization of Tf-PEM/L

The preparation of PE-MEs is based on our previous report (Chen et al., 2024b). PTX (10 mg) was firstly dissolved in mixture of *β*-elemene (80 mg) and 1944 CS (320 mg). After 2 h mixing, PEG 400 (150 mg) and HS 15 (450 mg) were then added and mixed for 2 more hours followed by slowly adding deionized water to 10 mL to obtain PE-MEs. The preparation method of Tf-PEM/L is as follows: 18.00 mg of DPPC, 1.60 mg of S-lysoPC, and 4.00 mg of DSPE-mPEG_2k_ were ultrasonically dissolved in 10 mL of CHCl_3_ using a 20 kHz probe sonicator at 50 W for 10 min (Chen et al., 2018). Using a rotary evaporator, the PE-MEs prepared in the previous step was hydrated at 50 °C under pressure for 3 h to obtain PEM/L. Finally, 1 mL of PEM/L was dispersed in 5 mL of deionized water, and 0.55 mg of Tf, 0.47 mg of NHS, and 0.74 mg of EDC were added. The reaction was carried out for 8 h to obtain Tf-PEM/L and followed by using flowing water for dialysis for 48 h to remove unreacted EDC and NHS. The particle size and zeta potential of Tf-PEM/L were measured using a dynamic light scattering (DLS) laser particle size analyzer (Nano-Z, Malvern, UK). The morphology of Tf-PEM/L was observed using a transmission electron microscope (TEM, JEM-2100F, JEOL, Japan) (Chen et al., 2020).

### 3.3 Encapsulation Efficiency (EE) and Loading Efficiency (LE) of Tf-PEM/L

Chromatographic conditions: Waters-C18 column (4.6 mm × 250 mm, 5 μm); Mobile phase: CH_3_CN-CH_3_OH-H_2_O in a ratio of 30: 40: 30; Detection wavelength set at 227 nm; Flow rate maintained at 1.0 mL/min (Chen et al., 2024a).

The calculations for EE and LE were conducted as follows:

$$\mathrm{EE}\;\left(\%\right)=W_{\text{encapsulated}\;\mathrm{PTX}}/W_{\text{total}\;\mathrm{PTX}}\times 100$$

$$\mathrm{LE}\;\left(\%\right)=W_{\text{encapsulated}\;\mathrm{PTX}}/W_{\text{weight of the freeze}-\text{dried}\;\mathrm{Tf}-\mathrm{PEM}/L}\times 100$$

### 3.4 In vitro drug release

Based on our prior report, the cumulative release profile of Tf-PEM/L was meticulously assessed utilizing high-performance liquid chromatography (HPLC). Specifically, 1 mL of Tf-PEM/L was ensconced within a dialysis bag (10 kDa molecular weight cutoff) and subsequently incubated in PBS at pH 7.4, with a total volume of 200 mL, under controlled conditions at temperatures of 37 °C and 42 °C while maintaining a rotational speed of 60 rpm.

### 3.5 Cell Culture

A549 cells were meticulously cultivated in DMEM enriched with 10% FBS (*v*/v), alongside 1% penicillin (100 IU/mL) and streptomycin (100 μg/mL). This process was conducted under controlled conditions of 37 °C, maintaining a humidity level of 95%, and an atmosphere comprising 5% CO_2_ (Ghorbanian et al., 2023; Zhang et al., 2024).

### 3.6 Intracellular delivery and cellular uptake

In celluar uptake study, A549 cells at density of 5 × 10^5^ were inoculated into 6-well plates and incubated for a duration of 24 h. Subsequently, A549 cells were treated with FITC, FITC-MEs, FITC-M/L, Tf-FITC-M/L at 1 mL per well, each containing a concentration of 10 μM (FITC concentration), over time intervals of 2, 4, and 6 h respectively. The fluorescence intensities associated with each experimental condition were precisely quantified using flow cytometry (BD FACSVerse, New Jersey, USA) (Miceli et al., 2022; Huang et al., 2024).

A549 cells were seeded in 6-well plates (2 × 10^5^) and allowed to adhere. A549 cells were pretreated with specific inhibitors including transferrin (1 mg/mL; clathrin-mediated endocytosis ligand competition), genistein (54 μg/mL; caveolae-mediated endocytosis inhibition), NH₄Cl (535 μg/mL; lysosomal acidification blockade), amiloride (133 μg/mL; macropinocytosis suppression), and sucrose (154 mg/mL; clathrin-mediated endocytosis inhibition), with a 4 °C inhibition group included as control. FITC, FITC-MEs, FITC-M/L and Tf-FITC-M/L were then incubated with the treated cells for 4 h, followed by quantitative analysis of intracellular fluorescence intensity using flow cytometry.

In the intracellular delivery investigation, A549 cells were seeded into laser confocal glass dishes at a density of 2 × 10^5^. All treatments including FITC, FITC-MEs, FITC-M/L and Tf-FITC-M/L were administered at a 1 mL per well with a reduced concentration of 5 μM (FITC concentration) for an exposure period of 2 h. Following this incubation period, Lysotracker Red was introduced and allowed to permeate for an additional 30 min. Ultimately, the cellular dynamics were meticulously examined and captured utilizing confocal laser scanning microscopy (Leica, TCS SP8, Germany).

### 3.7 Cytotoxicity

A549 cells at density of 5 × 10^3^ were harvested and subsequently seeded into 96-well plates, followed by a 24-h incubation period. All treatments involving PTX at concentrations ranging from 0.675 μg/mL to 20 μg/mL were categorized into distinct groups: PTX, PTX + *β*-elemene, PE-MEs, PEM/L and Tf-PEM/L. After the incubation period of 24 h, CCK8 assay was introduced and allowed to incubate for an additional 2 h at 37 °C. The absorbance (A) values were quantified using a microplate reader set to detect wavelengths at 450 nm.

### 3.8 Cell apoptosis induction

The induction of apoptosis in A549 cells by Tf-PEM/L was assessed utilizing the BestBio® Reagent Apoptosis Kit (BestBio, China). A549 cells at a density of 2 × 10^5^ were plated in six-well plates. Prior to the subsequent experimental procedures, all treatment groups were standardized to a uniform PTX concentration of 2 μg/mL before undergoing a subsequent incubation for 12 h. The overall apoptosis rate in A549 cells was assessed using flow cytometry.

### 3.9 Xenograft tumor models

In accordance with our previous report, the described procedure involves establishing an A549 xenograft tumor model in mice by subcutaneously injecting an A549 cell suspension containing approximately 2 × 10^7^ cells into the right hind limb. Tumor growth is then monitored by measuring the tumor size using vernier calipers and calculating the tumor volume with the formula:

$$V=\left(L\times W^{2}\right)/2$$

Where: V = Tumor volume.

L = Vertical length of the tumor.

W = Vertical width of the tumor.

### 3.10 In vivo imaging

Upon reaching a tumor volume of approximately 120 mm^3^, nude mice bearing A549 xenografts were randomly allocated into 4 groups: DiD, DiD-MEs, DiD-M/L, and Tf-DiD-M/L. An intraperitoneal injection comprising 0.2 mL each of DiD formulations was administered (DiD at a dosage equivalent to 30 μg/mL).

An in vivo imaging system (PerkinElmer IVIS Lumina LT; USA) facilitated the acquisition of near-infrared images following isoflurane anesthesia administration. Fluorescence post-administration was captured via region-of-interest (ROI) functionality during designated delivery intervals. Following 12 h post-treatment observation period all nude mice subjects underwent euthanasia, fluorescence images of major normal organs (heart, liver, spleen, lungs, and kidneys) as well as tumor tissues were collected using IVIS (Thouvenel et al., 2023; Xue et al., 2024).

### 3.11 Antitumor efficacy and systemic safety

Thirty A549 xenograft-bearing nude mice, each exhibiting an average tumor volume of 140 mm^3^, was subjected to subcutaneous injections every two days with PTX, PTX + *β*-elemene, PE-MEs, PEM/L and Tf-PEM/L at a concentration of 4 mg/kg (PTX). Tumor volume and body weight were meticulously recorded on a daily basis. After the completion of the administration period, blood samples were carefully collected from the ocular region of each nude mouse. The mice were humanely euthanized, and a range of critical tissues, including the heart, liver, spleen, lungs, kidneys-as well as tumor specimens, were systematically harvested. Subsequent procedures included paraffin embedding and histopathological sectioning accompanied by hematoxylin and eosin (HE) staining (Yang et al., 2024; Kalčec et al., 2023). All collected blood samples were employed for the comprehensive analysis of key cytokines and chemokines, including Interferon-γ (IFN-γ), tumor necrosis factor-α (TNF-α), transforming growth factor β_1_ (TGF-β_1_), and interleukin-2 (IL-2).

### 3.12 Data analysis

All data were subjected to thorough analysis using GraphPad Prism Software 9 and were presented as mean ± standard deviation (SD). Statistical significance is indicated by ^⁎^*P* < 0.05, reflecting a significant difference; ^⁎⁎^*P* < 0.01 denotes extreme significant difference.

## 4 Results and discussion

### 4.1 Preparation and Characterization of Tf-PEM/L

**Fig. 1:**
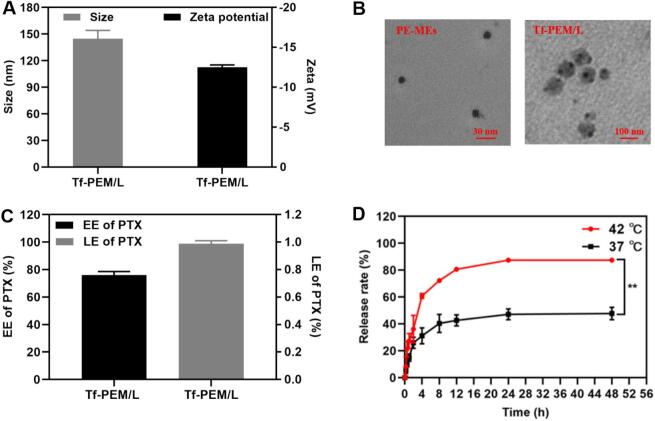
Characterization of Tf-PEM/L. (A) Particle size and zeta potential of Tf-PEM/L(n = 3). (B) TEM images of PE-MEs and Tf-PEM/L. (C) Encapsulation efficiency and drug loading efficiency of Tf-PEM/L(n = 3). (D) Release profile of Tf-PEM/L within 37 °C and 42 °C(n = 3). ^⁎⁎^*P* < 0.01.

PE-MEs was successfully encapsulated within liposomes and further modified with transferrin to fabricate the Tf-PEM/L. The Tf-PEM/L exhibited an average number-weighted particle size approximately (144.76 ± 9.34) nm, a polydispersity index of (0.19 ± 0.03), and a zeta potential of (−12.52 ± 0.28) mV (Fig. 1A and Table 1**)**. In particular, TEM images of Tf-PEM/L indicated that the structure of PE-MEs (∼10 nm) could be encapsulated within liposome intracavity (∼140 nm), indicating that PE-MEs can be successfully encapsulated within liposomes using the film dispersion method. Consequently, Tf-PEM/L demonstrated the ability to achieve two optimal particle sizes simultaneously, which can realize the transformation of large particle size with accumulation advantage to small particle size with deep penetration advantage under stimulation of tumor microthermal environment (Fig. 1B). A particle size of <200 nm enables the nanocarrier to exploit the Enhanced Permeability and Retention (EPR) effect, facilitating passive tumor targeting via extravasation through leaky tumor vasculature and prolonged retention in the tumor microenvironment. The EE of PTX in Tf-PEM/L was (76.01 ± 0.03)%, and meanwhile the LE of PTX was calculated at (0.99 ± 0.02)% (Fig. 1C)**.** In this study, the release profile of PTX was evaluated using a classical dialysis method in vitro. After 24 h of incubation in PBS at 42 °C (pH 7.4), the release rate of PTX from Tf-PEM/L was observed to be (87.44 ± 1.61)%, which was approximately 1.83-fold faster than that observed at 37 °C (Fig. 1D). Differences in release behavior between 37 °C and 42 °C offer the possibility that Tf-PEM/L could be triggered to release PTX under the stimulation of tumor microthermal environment.

**Table 1:** Average particle size PDI and Zeta Potential of Tf-PEM/L (*n* = 3, ^−^x ± s) (Measured by DLS).

Liposomes	Size (nm)	Polydispersity Index	Zeta Potential(mV)
Tf-PEM/L	144.77 ± 9.34	0.19 ± 0.03	−12.52 ± 0.28

### 4.2 Intracellular delivery of Tf-PEM/L

**Fig. 2:**
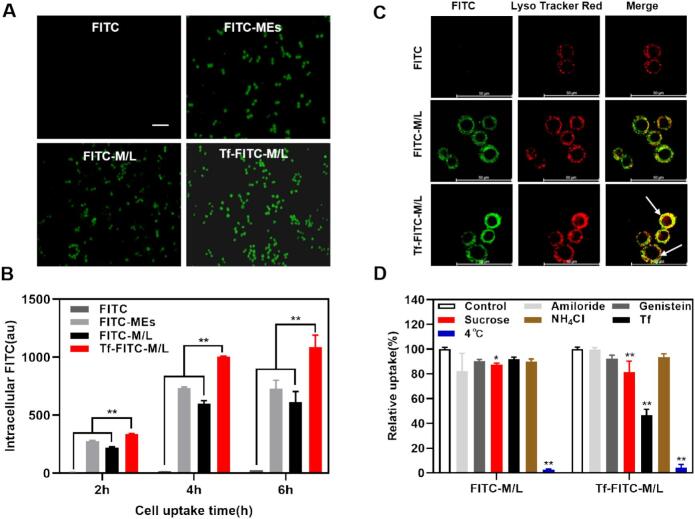
Cellular uptake. (A) Fluorescent images of A549 cells incubated with FITC, FITC-MEs, FITC-M/L, and Tf-FITC-M/L for 4 h. Scale bar: 100 μm. (B) Mean fluorescence intensity of A549 cells were analyzed by flow cytometry after incubation with preparations for 2, 4 and 6 h (n = 3). ^⁎⁎^*p* < 0.01. (C) Intracellular delivery of FITC, FITC-MEs, FITC-M/L and Tf-FITC-M/L within A549 cells observed using CLSM. Scale bar: 50 μm. (D) Relative uptake efficiency of FITC-M/L and Tf-FITC-M/L after pretreating A549 cells with different endocytosis inhibitors (n = 3). ^⁎^*P* < 0.05, ^⁎⁎^*p* < 0.01.

We meticulously evaluated the cellular uptake of various PTX formulations in a comprehensive study focused on internalization and intracellular delivery (Chibh et al., 2022). In comparison to free FITC, the intracellular fluorescence exhibited by FITC-MEs, FITC-M/L and Tf-FITC-M/L was markedly more pronounced. Specifically, the fluorescence intensity in A549 cells increased sequentially across the groups, with Tf-FITC-M/L displaying the highest fluorescence signal. This trend clearly suggests that nanoparticles including FITC-MEs, FITC-M/L and Tf-FITC-M/L enhanced the endocytosis of A549 cells, with Tf-mediated targeting further boosting cellular uptake efficiency. (^⁎⁎^*p* < 0.01) (Fig. 2A). The cellular uptake of FITC-MEs, FITC-M/L and Tf-FITC-M/L attained a plateau in uptake after a duration of 6 h after treatment. In particular, the fluorescence intensity of Tf-FITC-M/L uptake by A549 cells was measured at (1085.67 ± 105.08), representing a remarkable 1.77-fold increase compared to that of FITC-M/L, which recorded (613.33 ± 91.54) after a treatment duration of 6 h, indicating that transferrin modification enhanced its internalization (Fig. 2B) (Chen et al., 2023a）

Laser confocal microscopy was employed to meticulously investigate the intracellular delivery of FITC, FITC-MEs, FITC-M/L and Tf-FITC-M/L within A549 cells. FITC was adopted to label FITC-MEs, FITC-M/L and Tf-FITC-M/L with green fluorescence. LysoTracker Red was employed to fluorescently label endosomes and lysosomes. Notably, A549 cells treated with Tf-FITC-M/L exhibited a striking yellow fluorescence, indicating that Tf-FITC-M/L may be sequestered within the endosomal and lysosomal compartments (Fig. 2C) (Chen et al., 2023a; Chen et al., 2024b).

To elucidate the cellular uptake mechanisms in A549 cells, a range of specific uptake inhibitors including transferrin, ammonium chloride, genistein, amiloride and sucrose were systematically employed. Additionally, pre-incubation at 4 °C was utilized to effectively obstruct the endocytic pathways within A549 cells (Chen et al., 2023b; Chen et al., 2024a). The results indicated that following incubation with Tf-FITC-M/L, cellular uptake was significantly attenuated in the presence of sucrose and transferrin, suggesting that Tf-FITC-M/L was internalized via clathrin-mediated endocytosis and pathways mediated by transferrin receptor. Sucrose disrupts clathrin-mediated endocytosis by increasing the osmolarity of the extracellular environment which prevents the formation of clathrin-coated pits on the cell membrane-a key step for this endocytic pathway. Meanwhile, the internalization of FITC-M/L and Tf-FITC-M/L was significantly inhibited by pre-incubation at 4 °C, indicating that the internalization of FITC-M/L and Tf-FITC-M/L is in the manner of energy-dependent (Fig. 2D) (Wang et al., 2022a).

### 4.3 Antiproliferative efficacy in vitro

**Fig. 3:**
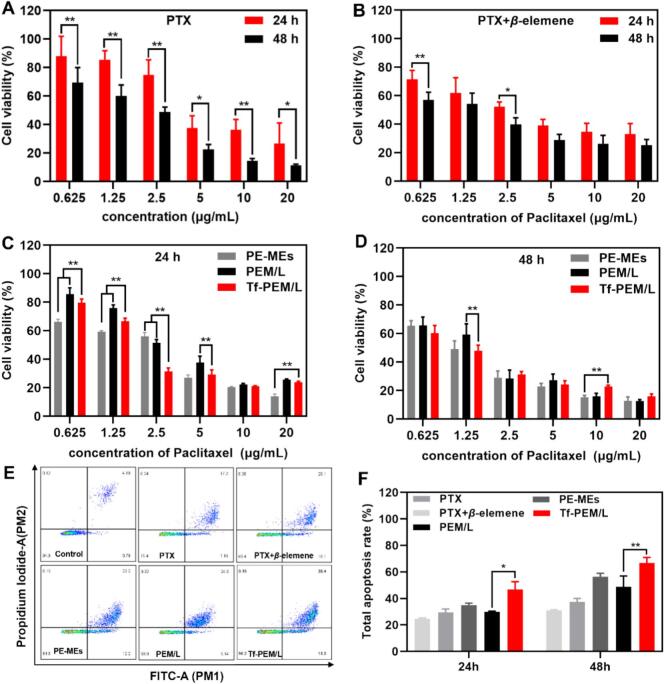
Cell apoptosis and cytotoxicity. (A) and (B) Cytotoxicity of PTX and PTX+ *β*-elemene treatments against A549 cells for 24 h and 48 h(*n* = 5). ^⁎^*P* < 0.05, ^⁎⁎^*P* < 0.01. Cytotoxicity of PE-MEs, PEM/L and Tf-PEM/L against A549 cells for (C) 24 h and (D) 48 h(n = 5). ^⁎⁎^*P* < 0.01. (E) Apoptosis ratio of A549 cells treated with various PTX treatments at concentration of 3 μg/mL for 24 h. (F) The quantitative analysis of apoptosis rate of A549 cells treated(n = 3). ^⁎^*P* < 0.05, ^⁎⁎^*P* < 0.01.

To comprehensively evaluate the synergistic anti-tumor efficacy of multi-component combinations against A549 cells, we conducted a detailed investigation into the cytotoxic effects of PTX, PTX + *β*-elemene, PE-MEs, PEM/L, and Tf-PEM/L. Using the CCK8 assay, we assessed cell viability at two time points, 24 h and 48 h, across a range of PTX concentrations spanning from 0.625 μg/mL to 20 μg/mL. PTX primarily involves irreversible covalent binding to tubulin, thereby specifically stabilizing microtubule polymer structures and effectively inhibiting the depolymerization dynamics. This interaction induces sustained arrest of the cell cycle at the G2/M phase checkpoint, subsequently activating a cascade of apoptotic signaling pathways through the mitochondrial pathway, ultimately leading to programmed cell death in tumor cells (Tian et al., 2023). After 24 h of treatment, the IC_50_ of PTX, PTX + *β*-elemene, PE-MEs, PEM/L and Tf-PEM/L were (5.274 ± 2.098) μg/mL, (3.129 ± 1.025) μg/mL, (2.005 ± 0.881) μg/mL, (3.310 ± 1.123) μg/mL and (2.053 ± 1.228) μg/mL, respectively. After 48 h of treatment, the IC_50_ of PTX, PTX + *β*-elemene, PE-MEs, PEM/L and Tf-PEM/L were (1.812 ± 0.518) μg/mL, (1.221 ± 0.645) μg/mL, (1.173 ± 0.348) μg/mL，(1.404 ± 0.619) μg/mL and (1.031 ± 0.429) μg/mL, respectively (Fig. 3A-3D)**.** The CCK8 assay also confirmed the advantage of PE-MEs with small particle size in deep penetration.

### 4.4 Cell apoptosis induction

To validate the efficacy of our innovative combined anti-cancer therapy strategy, we utilized Annexin V-PE/7-AAD staining to quantitatively assess apoptosis induction in A549 cells treated with PTX, PTX + *β*-elemene, PE-MEs, PEM/L, and Tf-PEM/L. A549 cells were subjected to various PTX formulations at a concentration of 3 μg/mL, with subsequent evaluation of apoptosis occurring after 24 h and 48 h of incubation (Saeed et al., 2022). The reported apoptosis percentages for all treatment groups correspond to the sum of early and late apoptotic cells. The apoptosis rates of A549 cells in PTX，PTX + *β*-elemene，PE-MEs，PEM/L and Tf-PEM/L were (24.64 ± 0.73)%, (29.50% ± 2.52)%, (34.93 ± 1.55)%, (29.98 ± 0.33)% and (46.67 ± 5.93)% after treatment for 24 h, respectively. The apoptosis rates of A549 cells in PTX, PTX + *β*-elemene, PE-MEs, PEM/L and Tf-PEM/L were (30.87 ± 0.64)%, (37.40 ± 2.69)%, (56.47 ± 2.60)%, (48.77 ± 8.17)% and (66.90 ± 4.02)% after treatment for 48 h, respectively (Fig. 3E and F; Fig. S1). Notably, Tf-PEM/L induced 1.9- and 1.8-fold higher apoptosis rates than free PTX at 24 h and 48 h, respectively. Our research validates that Tf-PEM/L can conspicuously induce extensive apoptosis of A549 cells, indicating that Tf-PEM/L is expected to obtain better anti-tumor efficacy in vivo (Niu et al., 2023)).

### 4.5 Biodistribution

To clarify the in vivo distribution of Tf-PEM/L using near-infrared imaging, we labeled PE-MEs, PEM/L, and Tf-PEM/L with DiD, generating DiD-MEs, DiD-M/L and Tf-DiD-M/L, respectively (Zajdel et al., 2023; Xu et al., 2023).

**Fig. 4:**
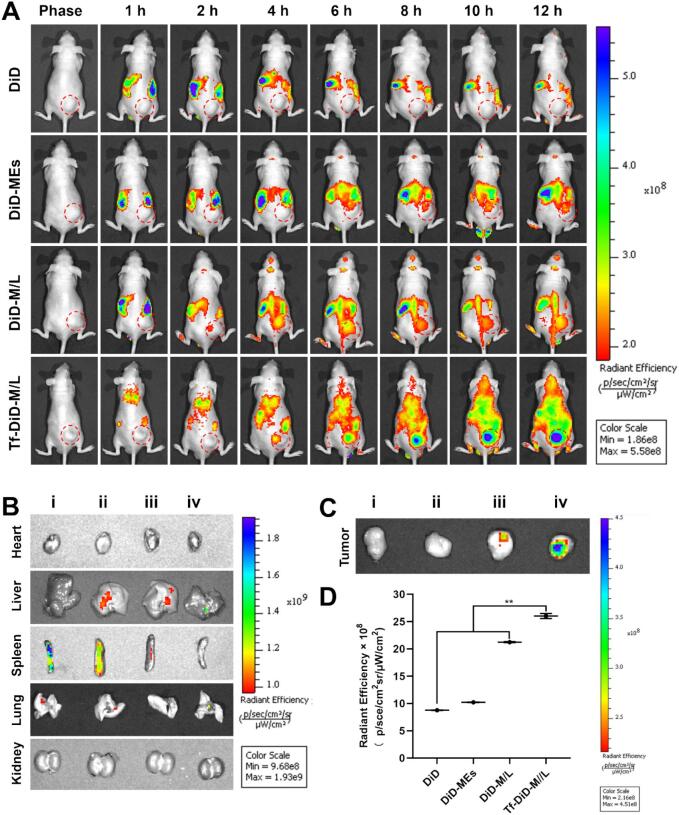
Investigation of biodistribution. (A) Distribution of NIR signal in mice treated with different DiD-labeled formulations at predetermined intervals. (B) Fluorescence distribution in different normal organs at 12 h post-injection. (C) Fluorescence images of tumor and brain tissues ex vivo at 12 h post-injection. (D) Quantitative analysis of fluorescence in the tumor tissues 12 h after administration (n = 3). ^⁎⁎^*P* < 0.01. (i), (ii), (iii), (iv) represent DiD, DiD-MEs, DiD-M/L, Tf-DiD-M/L.

In the DiD group, no significant fluorescence aggregation was observed at the tumor site over the 1–12 h period. In contrast, treatment with Tf-DiD-M/L resulted in markedly enhanced fluorescence accumulation at the tumor site during the same time frame. It is noteworthy that a strong and distinct near-infrared signal was consistently detected at the tumor site throughout the entire observation period following Tf-DiD-M/L treatment (Fig. 4A) (Janssen et al., 2024). This enhanced tumor targeting can be attributed to the specific recognition between transferrin on the nanocarrier surface and TfR overexpressed on NSCLC cells, as well as the passive targeting effect mediated by the EPR effect (Wang et al., 2025) Our findings confirm that TfR-mediated active targeting is a reliable strategy to enhance the tumor-selective delivery of chemotherapeutic agents. DiD-MEs, DiD-M/L and Tf-DiD-M/L were mainly aggregated in the liver after treatment for 12 h, indicating that Tf-DiD-M/L was mainly captured by the reticuloendothelial system (RES) (Fig. 4B). It is worth noting that, consistent with the biodistribution characteristics of nanocarriers, minor liver accumulation of Tf-PEM/L was observed, which represents a typical clearance route mediated by the RES—a common physiological process for nanomedicines. Tumor tissues were collected for quantitative and qualitative analysis of fluorescence intensity to evaluate potential tumor targeting ability (Fig. 4C). Tf-DiD-M/L displayed distinct biodistribution characteristics when contrasted with DiD-MEs and DiD-M/L (Fig. 4D). Remarkably, the tumor sites in mice treated with Tf-DiD-M/L displayed the highest fluorescence intensity, suggesting that transferrin-based active targeting facilitates efficient tumor localization. It is worth noting that Tf-DiD-M/L possessed remarkable brain-targeting ability and holds certain potential for the treatment of brain metastatic lung cancer (Fig. S2) (Nardone et al., 2023; Adua et al., 2022; Salari et al., 2024).

### 4.6 Evaluation of antitumor efficacy

**Fig. 5:**
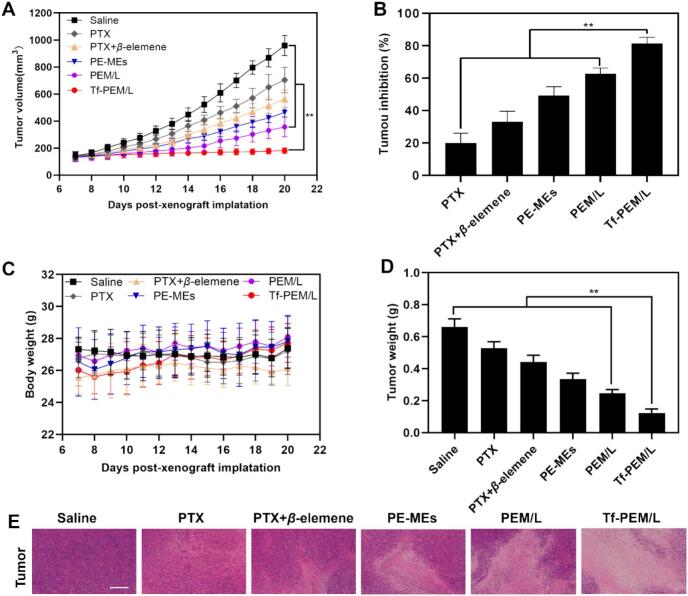
Antitumor efficacy in vivo. (A) Changes in tumor volumes of mice treated with different treatments (n = 5). ^⁎⁎^*P* < 0.01. (B) Inhibition rates of tumor growth in mice treated with different formulations at day 22 post-xenograft implantation (n = 5). ^⁎⁎^*P* < 0.01. (C) Alterations in body weights of mice during treatments (n = 5). ^⁎⁎^*P* < 0.01. (D) Tumor weights of mice treated with various formulations at the end of the observed period (n = 5). ^⁎⁎^*P* < 0.01. (E) HE-stained images of the tumor slides of mice after different therapies. The scale bar is 200 μm.

In order to verify the active targeting of transferrin-modified nanocarrier system after modification, saline, PTX, PTX + *β*-elemene, PE-MEs and PEM/L was adopted as controls to study the anti-tumor efficacy of Tf-PEM/L by using A549 tumor-bearing nude mice (Li et al., 2021). We treated the A549 tumor-bearing nude mice every two days by intraperitoneal injection of various PTX treatments with a dose of 10 mg/kg. Compared with PTX, PTX + *β*-elemene, PE-MEs and PEM/L, Tf-PEM/L can significantly inhibited tumor growth, which may be attributed to enhanced drug accumulations at the tumor sites by transferrin modification, is closely correlated with its superior tumor targeting ability revealed by biodistribution studies (Fig. 5A and S3). The increased drug accumulation in tumor tissues ensures sufficient local drug concentration to exert synergistic anti-tumor effects of paclitaxel and *β-*elemene. The tumor inhibition rate of Tf-PEM/L was (81.36 ± 3.87)%, representing increases of 4.05-fold, 2.45-fold, 1.65-fold and 1.31-fold higher over than that of PTX, PTX + *β*-elemene, PE-MEs and PEM/L, respectively (Fig. 5B). Throughout the observation period, there was only slight fluctuation in body weight after treated with various PTX treatments (Fig. 5C). After treatment with Tf-PEM/L had the lowest tumor weight among various PTX treatments (Fig. 5D). Furthermore, Tf-PEM/L showed the largest area of necrosis in HE-stained images of A549 xenograft tumors in nude mice (Fig. 5E) (Kang et al., 2024). In summary, Tf-PEM/L exhibited synergistic anti-tumor proliferation ability via deep penetration based on multi-component small-sized mediation and active targeting advantage by transferrin modification (Wang et al., 2025).

### 4.7 Evaluation of systemic safety

**Fig. 6:**
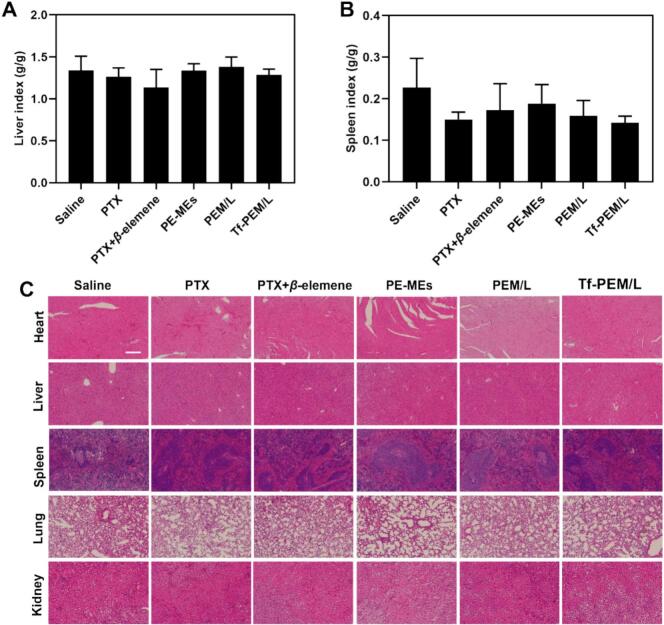
Evaluation of safety in vivo. (A) Liver index and (B) spleen index of mice treated with different formulations (n = 5). ^⁎⁎^*P* < 0.01. (C) Pathological sections of HE-stained normal organs after different treatments. The scale bar is 200 μm.

In this study, the A549 tumor-bearing nude mice model and liver-spleen index were utilized as indicators to determine the safety profiles of various PTX treatments (Kudo et al., 2023). In comparison with the saline group, the liver-spleen index remained largely unchanged in all PTX treatment groups, with no evidence of liver or spleen injury (Fig. 6A and B). Additionally, H&E staining studies among various organs were utilized to further evaluate the safety of various PTX treatments. No notable changes in liver and spleen index ratios were detected by comparing with the saline group. Moreover, all PTX treatments showed no observable abnormalities in the heart, liver, spleen, kidney, or lung tissues (Fig. 6C) (Chen and Wen, 2022).

In conclusion, evaluation of systemic safety studies demonstrated that Tf-PEM/L exhibit neither hepatotoxicity nor nephrotoxicity, and meanwhile it histologically confirming both efficacy and safety of various PTX treatments. At the same time, throughout the treatment period, no signs of acute distress or abnormal behavior were observed in the experimental animals.

### 4.8 Detection of cytokines

**Fig. 7:**
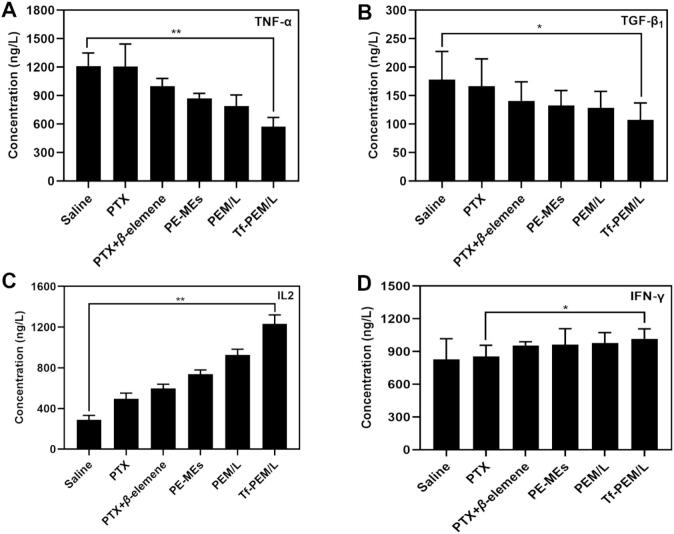
Detection of cytokines. (A) TNF-α (B) TGF-β1 (C) IL-2 (D) IFN-γ in serum of various PTX treatments (n = 5). ^⁎⁎^*p* < 0.01.

The role of cytokines is pivotal in the initiation and progression of tumorigenesis (Cai et al., 2022). The inflammatory cytokine TNF-α serves a central function in coordinating immune and inflammatory responses mediated by macrophages and monocytes (Wang et al., 2022b). In contrast, TGF-β_1_ serves as a transformative growth factor that not only fosters tumorigenesis but also exerts immunosuppressive effects that significantly facilitate tumor development (Nan et al., 2022). In our study, Tf-PEM/L markedly attenuated TNF-α and TGF-β_1_ expression levels in comparison to the saline group, pointing to its potential role in inhibiting tumor proliferation through its suppressive action on TNF-α and TGF-β_1_ expression (Fig. 7A and B). Furthermore, IFN-γ is known to activate M1-type tumor-associated macrophages (TAMs) while concurrently diminishing their population by inhibiting angiogenesis pathways; this mechanism effectively obstructs the proliferation of neoplastic cells (Zhuang et al., 2023). Additionally, IL-2 holds considerable significance within the immune response framework (Muhammad et al., 2023). Notably, following treatment with Tf-PEM/L, we observed elevated expression levels of both IFN-γ and IL-2 relative to those in the saline group, underscoring that Tf-PEM/L pronounced efficacy in curtailing TAMs formation (Fig. 7C and D).

## 5 Conclusions

In conclusion, this study successfully developed transferrin-modified multi-component liposomes (Tf-PEM/L) by encapsulating small-sized PEM and systematically evaluated their anti-NSCLC efficacy and safety. The optimized Tf-PEM/L efficiently accumulated in tumors via Tf-mediated active targeting combined with the Enhanced Permeability and Retention (EPR) effect. After tumor accumulation, Tf-PEM/L released small-sized PEM for deep tumor penetration, and the co-delivered paclitaxel and *β*-elemene exerted synergistic anti-NSCLC effects. Collectively, our study establishes a groundbreaking strategy for combined NSCLC therapy, underscoring its potential to advance treatment outcomes.

## CRediT authorship contribution statement

**Yunyan Chen:** Methodology, Investigation, Funding acquisition, Conceptualization. **Ziwei Zhang:** Investigation. **Rui Xiong:** Investigation. **Yuqing Cao:** Investigation. **Qian Liu:** Investigation, Funding acquisition.

## Declaration of competing interest

The authors declare the following financial interests/personal relationships which may be considered as potential competing interests: Yunyan Chen reports financial support was provided by National Natural Science Foundation of China. Yunyan Chen reports a relationship with Anhui Provincial Natural Science Foundation that includes: funding grants. If there are other authors, they declare that they have no known competing financial interests or personal relationships that could have appeared to influence the work reported in this paper.

## Data Availability

The data that has been used is confidential.

## Appendix A Supplementary data

Supplementary material Image 1
