# Supplementary material for: Transferrin-modified multicomponent liposomes encapsulating paclitaxel-loaded β-elemene microemulsion enhance therapeutic efficacy in non-small-cell lung cancer
Source: Int J Pharm X. 2026 Jan 12;11:100488. doi: 10.1016/j.ijpx.2026.100488 (PMC12830280; doi:10.1016/j.ijpx.2026.100488)
Supplement: Supplementary file 1 — Supplementary material [file mmc1.docx]

**Transferrin-modified multicomponent liposomes encapsulating paclitaxel-loaded *β*-elemene microemulsion enhance therapeutic efficacy in non-small-cell lung cancer**

Yunyan Chen ^a,1*^, Ziwei Zhang ^a,1^, Rui Xiong ^a^, Yuqing Cao ^a^, Qian Liu ^b^

a School of Pharmacy, Wannan Medical College, Wuhu 241002, China.

b The Affiliated Brain Hospital of Nanjing Medical University, Nanjing, 210029, China.

* Corresponding authors: Dr. Yunyan Chen

1 These authors contributed equally to this work.

School of Pharmacy, Wannan Medical College, Wuhu 241002, China.

Tel: +86 553 3932492

1. mail: [cyy1206@163.com](mailto:cyy1206@163.com)


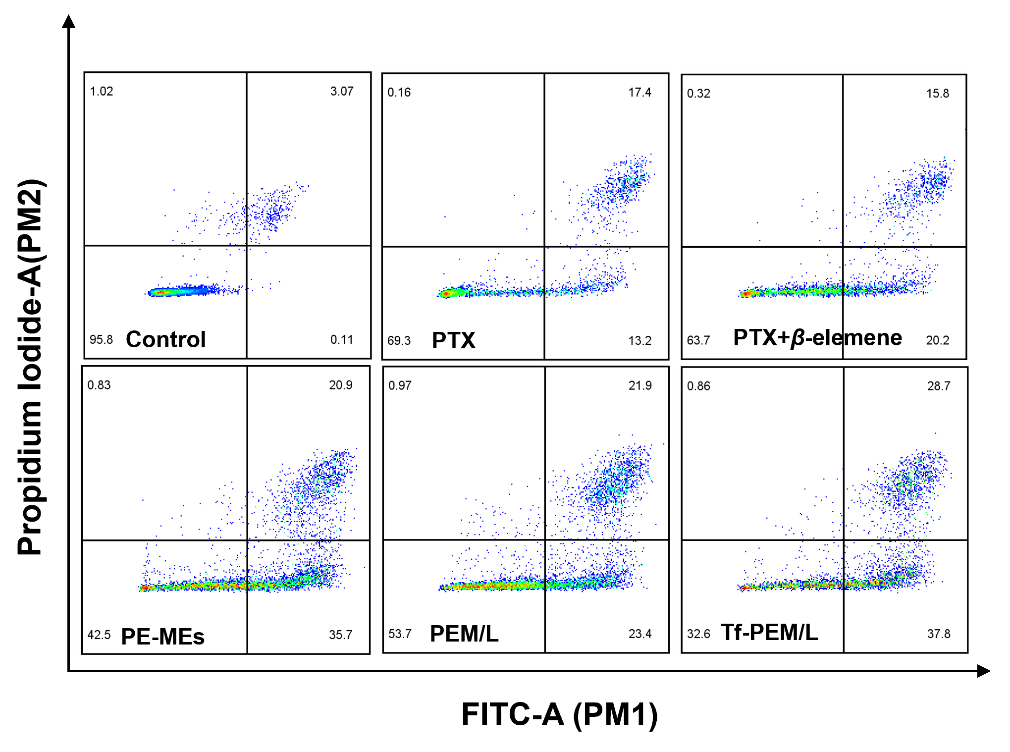


Fig. S1. Apoptosis ratio of A549 cells treated with various PTX treatments at concentration of 3 μg/mL, for 48 h.


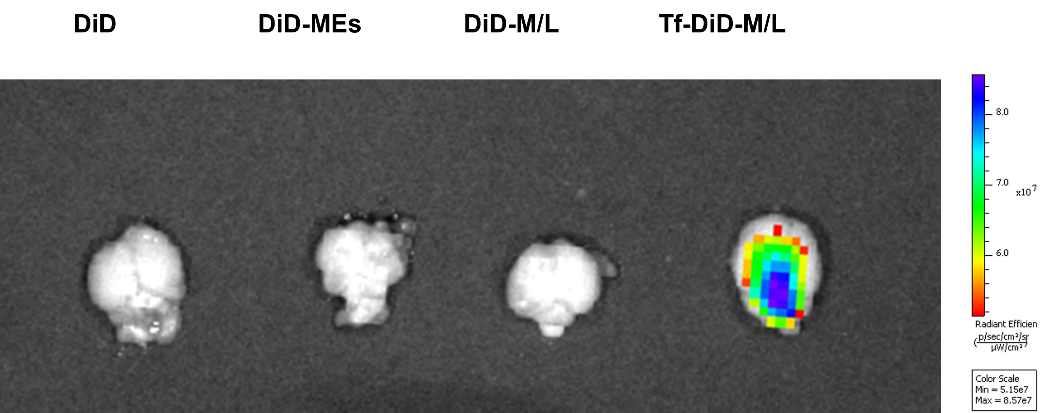


Fig. S2. Fluorescence images of tumor tissues at 12 h post-injection.


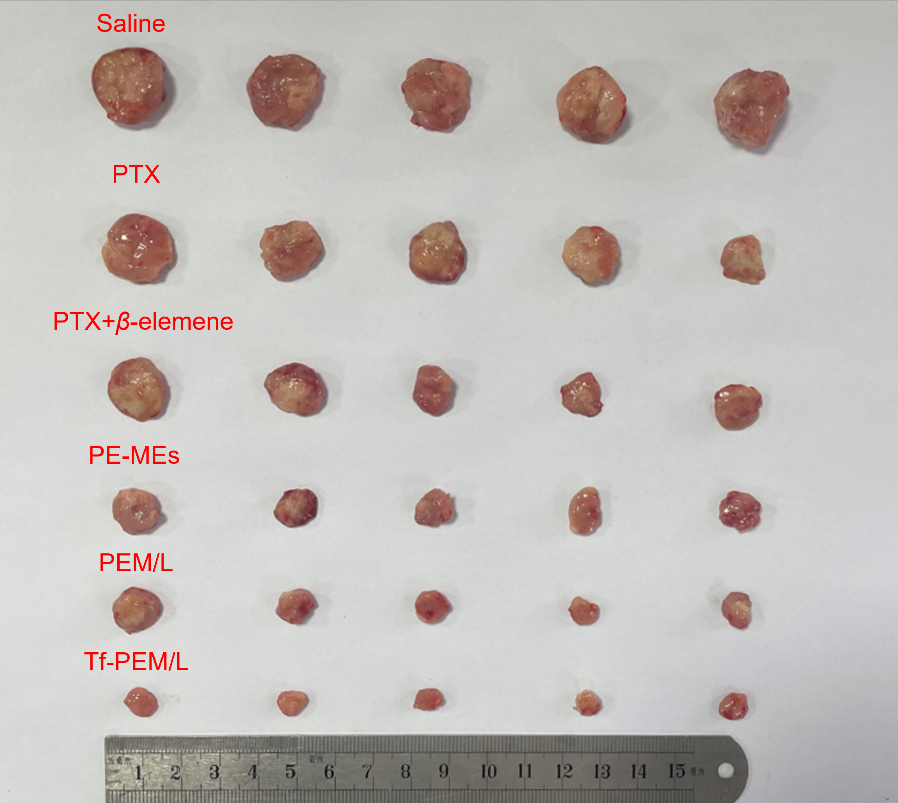


Fig. S3. The tumors of Saline, PTX, PTX+*β*-elemene, PE-MEs, PEM/L and Tf-PEM/L of mice (n=5).
